# Association of lipid profile biomarkers with breast cancer by molecular subtype: analysis of the MEND study

**DOI:** 10.1038/s41598-022-13740-x

**Published:** 2022-06-23

**Authors:** Anjali Gupta, Veeral Saraiya, April Deveaux, Taofik Oyekunle, Klarissa D. Jackson, Omolola Salako, Adetola Daramola, Allison Hall, Olusegun Alatise, Gabriel Ogun, Adewale Adeniyi, Omobolaji Ayandipo, Thomas Olajide, Olalekan Olasehinde, Olukayode Arowolo, Adewale Adisa, Oludolapo Afuwape, Aralola Olusanya, Aderemi Adegoke, Trygve O. Tollefsbol, Donna Arnett, Michael J. Muehlbauer, Christopher B. Newgard, H3 Africa Kidney Research Network, Samuel Ajayi, Yemi Raji, Timothy Olanrewaju, Charlotte Osafo, Ifeoma Ulasi, Adanze Asinobi, Cheryl A. Winkler, David Burke, Fatiu Arogundade, Ivy Ekem, Jacob Plange-Rhule, Manmak Mamven, Michael Mate-kole, Olukemi Amodu, Richard Cooper, Sampson Antwi, Adebowale Adeyemo, Titilayo Ilori, Victoria Adabayeri, Alexander Nyarko, Anita Ghansah, Ernestine Kubi Amos-Abanyie, Priscilla Abena Akyaw, Paul L. Kimmel, Babatunde L. Salako, Rulan S. Parekh, Bamidele Tayo, Rasheed Gbadegesin, Michael Boehnke, Robert Lyons, Frank Brosius, Daniel Clauw, Chijioke Adindu, Clement Bewaji, Elliot Koranteng Tannor, Perditer Okyere, Chuba Ijoma, Nicki Tiffin, Junaid Gamiedien, Friedhelm Hildebrandt, Charles Odenigbo, Nonyelun Jisieike-Onuigbo, Ifeoma Modebe, Aliyu Abdu, Patience Obiagwu, Ogochukwu Okoye, Adaobi Solarin, Toyin Amira, Christopher Esezobor, Muhammad Makusidi, Santosh Saraf, Victor Gordeuk, Gloria Ashuntangtang, Georgette Guenkam, Folefack Kazi, Olanrewaju Adedoyin, Mignon McCullough, Peter Nourse, Uche Okafor, Emmanuel Anigilaje, Patrick Ikpebe, Tola Odetunde, Ngozi Mbanefo, Wasiu Olowu, Paulina Tindana, Olubenga Awobusuyi, Olugbenga Ogedegbe, Opeyemi Olabisi, Karl Skorecki, Ademola Adebowale, Matthias Kretzler, Jeffrey Hodgin, Dwomoa Adu, Akinlolu Ojo, Vincent Boima, Tomi Akinyemiju

**Affiliations:** 1 Trinity College of Arts and Sciences, Duke University, Durham, NC USA; 2 Department of Population Health Sciences, School of Medicine, Duke University, 215 Morris Street, Durham, NC 27708 USA; 3 Department of Epidemiology, University of North Carolina Gillings School of Global Public Health, Chapel Hill, NC USA; 4 Divison of Pharmacotherapy and Experimental Therapeutics, University of North Carolina at Chapel Hill Eshelman School of Pharmacy, Chapel Hill, NC USA; 5 College of Medicine &, Lagos University Teaching Hospital, University of Lagos, Lagos, Lagos State, Nigeria; 6 Department of Pathology, School of Medicine, Duke University, Durham, NC USA; 7 Obafemi Awolowo University Teaching Hospital, Ile-Ife, Osun State Nigeria; 8 Unversity College Hospital, University of Ibadan, Ibadan, Oyo State, Nigeria; 9 Federal Medical Center, Ogun State, Abeokuta, Nigeria; 10 Our Lady of Apostle Catholic Hospital, Ibadan, Oyo State, Nigeria; 11 University of Alabama at Birmingham, Birmingham, AL USA; 12 College of Public Health, University of Kentucky, Lexington, KY USA; 13 Duke Molecular Physiology Institute, Duke University, Durham, NC USA; 14 Medical Center, University of Kansas, Kansas City, KS USA; 15 Duke Cancer Institute, School of Medicine, Duke University, Durham, NC USA; 16 Duke Global Health Institute, Duke University, Durham, NC USA; 17 Department of Medicine, Pediatrics and Institute of Child Health, University of Ibadan, Ibadan, Nigeria; 18 University of Ilorin, Ilorin, Nigeria; 19 University of Ghana Medical School, Accra, Ghana; 20 University of Nigeria, Nsukka, Enugu State Nigeria; 21 Basic Research Laboratory, Frederick National Laboratory for Cancer Research, National Cancer Institute, Frederick, MD USA; 22 Departments of Human Genetics, Internal Medicine and Pathology, University of Michigan, Ann Arbor, MI USA; 23 Obafemi Awolowo University, Ile-Ife, Nigeria; 24 University of Cape Coast, Cape Coast, Ghana; 25 Kwame Nkrumah University of Science and Technology, Kumasi, Ghana; 26 University of Abuja, Abuja, Nigeria; 27 Parkinson School of Health Sciences and Public Health, Loyola University, Chicago, IL USA; 28 Centre for Research on Genomics and Global Health, National Human Genome Research Institute, National Institutes of Health, Bethesda, MD USA; 29 Division of Nephrology, Boston Medical Center, Boston University School of Medicine, Boston, MA USA; 30 Noguchi Memorial Institute for Medical Research, University of Ghana, Accra, Ghana; 31 National Institute of Diabetes and Digestive and Kidney Disease, Bethesda, MD USA; 32 Department of Pediatrics, University of Toronto, Toronto, Canada; 33 Department of Pediatrics, Duke University Medical Center, Durham, NC USA; 34 University of Western Cape, Cape Town, South Africa; 35 Harvard Medical School, Harvard University, Boston, MA USA; 36 Nnamdi Azikiwe University Teaching Hospital, Nnewi, Nigeria; 37 Aminu Kano Teaching Hospital, Kano, Nigeria; 38 Delta State University Teaching Hospital, Warri, Nigeria; 39 Lagos State University Teaching Hospital, Lagos, Nigeria; 40 Lagos University Teaching Hospital, College of Medicine, University of Lagos, Lagos, Nigeria; 41 Usmanu Danfodiyo University Teaching Hospital, Sokoto, Nigeria; 42 University of Illinois at Chicago, Chicago, IL USA; 43 University of Yaoundé, Yaoundé, Cameroon; 44 University of Cape Town, Cape Town, South Africa; 45 New York University, New York City, NY USA; 46 Duke University, Durham, NC USA; 47 Technion-Israel Institute of Technology, Haifa, Israel; 48 School of Medicine, University of Kansas, Kansas City, KS USA

**Keywords:** Diagnostic markers, Breast cancer

## Abstract

There is conflicting evidence on the role of lipid biomarkers in breast cancer (BC), and no study to our knowledge has examined this association among African women. We estimated odds ratios (ORs) and 95% confidence intervals (95% CI) for the association of lipid biomarkers—total cholesterol, high-density lipoprotein (HDL), low-density lipoprotein (LDL), and triglycerides—with odds of BC overall and by subtype (Luminal A, Luminal B, HER2-enriched and triple-negative or TNBC) for 296 newly diagnosed BC cases and 116 healthy controls in Nigeria. Each unit standard deviation (SD) increase in triglycerides was associated with 39% increased odds of BC in fully adjusted models (aOR: 1.39; 95% CI: 1.03, 1.86). Among post-menopausal women, higher total cholesterol (aOR: 1.65; 95% CI: 1.06, 2.57), LDL cholesterol (aOR: 1.59; 95% CI: 1.04, 2.41), and triglycerides (aOR: 1.91; 95% CI: 1.21, 3.01) were associated with increased odds of BC. Additionally, each unit SD increase in LDL was associated with 64% increased odds of Luminal B BC (aOR 1.64; 95% CI: 1.06, 2.55). Clinically low HDL was associated with 2.7 times increased odds of TNBC (aOR 2.67; 95% CI: 1.10, 6.49). Among post-menopausal women, higher LDL cholesterol and triglycerides were significantly associated with increased odds of Luminal B BC and HER2 BC, respectively. In conclusion, low HDL and high LDL are associated with increased odds of TN and Luminal B BC, respectively, among African women. Future prospective studies can definitively characterize this association and inform clinical approaches targeting HDL as a BC prevention strategy.

## Introduction

Breast cancer (BC) in Nigeria, like in other West African countries and among Black patients in the United States (US), is characterized by disproportionately high rates of the triple-negative (TN) molecular subtype^1–3^. TNBCs are aggressive cancers, described by estrogen (ER), progesterone (PR), and human epidermal growth factor receptor 2 (HER2) negativity and associated with poor clinical outcomes^4,5^. Africa suffers from the highest age-standardized BC mortality rate globally^6^, and the past few decades have observed increasing BC incidence on the African continent^7^. An understanding of the risk factors contributing to the higher prevalence of TNBCs among women of African descent is crucial to the development of preventive interventions that may reduce the BC burden within this population. In addition to increasing BC incidence, the African continent has also experienced significantly increasing rates of obesity, diabetes, and dyslipidemia (abnormally elevated blood cholesterol or lipid levels), so called “diseases of affluence” due to globalization and the epidemiologic transition^8,9^. Prior studies have documented a positive association between measures of excess adiposity and BC incidence^10,11^, but none to our knowledge has examined specific biomarkers associated with dyslipidemia with BC risk by molecular subtype on the African continent.

Prior studies in the US, Europe and parts of Asia evaluating the relationship between serum lipids and risk of BC have been inconclusive, and several review papers have summarized published results on this topic. A recent systematic review of prospective studies reported an inverse association between biomarkers of total cholesterol and high-density lipoprotein (HDL) cholesterol and risk of breast cancer, but no significant associations with low-density lipoprotein (LDL) cholesterol^12^. This study noted significant heterogeneity among included studies for total cholesterol based on geographical location. The inverse association for HDL cholesterol was replicated in a separate systematic review which also reported a positive association for LDL cholesterol^13^. A third meta-analysis found that higher triglyceride levels, but not total cholesterol, HDL cholesterol or LDL cholesterol levels was inversely associated with BC risk^14^. It is worth noting that the majority of studies on this topic have been conducted among White populations in the United States and Europe. Studies among African American populations are limited and conflicting. While one study among African Americans in the United States found a statistically significant reduction in BC risk with high levels of total cholesterol and a significant increase in risk associated with low HDL cholesterol^15^; another study reported no significant association with total cholesterol^16^. Research on this topic deserves further study to more clearly elucidate the association between lipid biomarkers and BC risk. To our knowledge, studies on this topic have not been conducted in Nigeria or West Africa.

Importantly, few epidemiological studies have examined the association between lipids and BC molecular subtype. One study in Korea noted that low HDL cholesterol and high levels of triglycerides were associated with an increased risk of developing hormone receptor negative tumors^17^. Another study in Spain found that the risk of postmenopausal Luminal A BC significantly increased with higher circulating levels of triglycerides^18^. However, no study to our knowledge has examined this association among African women or in African American women, despite the higher risk of TNBC in these populations. To our knowledge, ours is the first study to evaluate the association between total cholesterol, LDL cholesterol, HDL cholesterol, and triglycerides with BC molecular subtypes among Nigerian women. Blood lipids are easily measurable markers that are routinely assessed in clinical practice. Thus, further insight on this relationship by molecular subtype may enable the development of preventative strategies that are well-suited to the Nigerian and African context.

## Methods

### Study design

The Mechanisms for Established and Novel Risk Factors for Breast Cancer in Women of Nigerian Descent (MEND) study has been previously described in detail^19^. Briefly, MEND enrolled newly diagnosed BC patients from four hospitals in southwestern Nigeria. At each hospital site, a trained nurse explained the study requirements to suspected BC patients during their clinical visits. Interested participants were evaluated for eligibility. Reasons for exclusion included an inability to communicate in English to complete the required baseline survey, prior diagnosis and/or treatment for cancer, and other medical conditions that may have interfered with participation in the study. All study participants gave written and verbal informed consent, and then completed a questionnaire that covered information on sociodemographic characteristics, reproductive history, and past personal and family history of cancer. Anthropomorphic measurements were taken, and blood samples and tumor biopsy samples were collected. All samples were obtained at the time of biopsy prior to receipt of any surgery, chemotherapy, or radiation treatment. After collection and processing, tissue and blood samples were stored in −80 °C freezers until shipment to the United States for assays and further analysis. For their participation in this study, participants received an N500 telephone recharge card (valued at US $1.50) in addition to the supplies necessary for their biopsy. Healthy controls were selected from a cohort of 4,000 healthy, community-based women recruited as part of the Human Heredity and Health (H3) Africa Chronic Kidney Disease (CKD) Case–Control study^20^. The CKD study recruited from Nigeria and Ghana between 2015 and 2017, overlapping with case recruitment. Controls were recruited from churches, communities, and business offices. The present analysis was restricted to controls recruited from Nigeria due to significant country-level differences in cholesterol. Recruitment of Nigerian controls occurred in the South-Western region of the country, overlapping with the case recruitment region. Extensive socio-demographic, clinical, family history and behavioral risk factor data was collected, and blood samples were collected and processed at clinical labs following a standardized protocol. Serum samples for cases and controls were assayed for lipid biomarkers at the Duke Molecular Pathology Institute at the same time, and the laboratory technician was blinded to case status. These procedures were approved by the Institutional Review Boards at Duke University and the participating hospitals. Among MEND cases, there were only 15 refusals and 1 withdrawal, and similarly low rates were observed among controls.

### Breast cancer cases and subtyping

BC diagnosis was ascertained either through pathology reports of clinical biopsy samples evaluated by a trained pathologist from the diagnosing hospital in Nigeria, or from biopsy samples that were shipped to the US for review by a trained US pathologist. If either indicated a cancer diagnosis, the sample was considered a confirmed case. Confirmed samples underwent immunohistochemistry in Nigeria as part of regular standard of care procedures, or at the Duke University BioRepository and Precision Pathology Center. Due to infrastructural limitations, it was not possible to complete immunohistochemistry within routine clinical care locally in Nigeria for all patients. If results from both sources were available, US typing was used as it constituted most of the available immunohistochemistry information on cases. Estrogen receptor (ER) and progesterone receptor (PR) status was scored using the Allred method^21,22^. The intensity of staining was categorized as 0 (none), 1 (mild), 2 (moderate), or 3 (strong), and the proportion of nuclear positivity was scored into 0 (0%), 1 (< 1%), 2 (1–10%), 3 (11–33%), 4 (33–66%) or 5 (67–100%). The numbers from these two scores were summed to positive (3–8) or negative (0–2). HER2 status was categorized as negative (scores = 0–1) or positive (score = 3) based on immunohistochemistry membrane staining; intense membrane staining of 30% of tumor cells constituted a positive result^23^. There were no equivocal (score = 2) results in our sample. Based on these categorizations, cancer subtype was determined: Luminal A (ER+ and/or PR+ /HER2-), Luminal B (ER+ and/or PR+ /HER2 +), TN (ER-/PR-/HER2-), or HER2 (ER-/PR-/HER2+). In all, there were 124 cases with available data on ER/PR/HER2 status for classification into a molecular subtype. There was no systematic selection of cases for subtyping. Cases with available molecular subtypes were similar to all cases by demographic, clinical, and reproductive characteristics.

### Measures

Measurements of total cholesterol, HDL, LDL, and triglycerides for cases and controls were performed using a Beckman DxC 600 clinical analyzer with assays that utilized standard reagents also from Beckman (Brea, CA). There was no systematic selection of participants for lipid measurements. Cases with available lipid results were similar to all cases by demographic, clinical, and reproductive characteristics. Following the joint harmonized criteria for metabolic syndrome and guidelines set by the National Cholesterol Education Program, high total cholesterol was defined as >200 mg/dL^24^; low HDL was defined as <50 mg/dL^25^; high LDL was defined as >100 mg/dL^24^; and high triglycerides was defined as >150 mg/dL^25^. In addition, lipid measures were specified as standard deviation (SD) change by subtracting the sample mean from individual measurements and dividing by the sample standard deviation. Other covariates included in analysis were staff assessed height, weight, blood pressure; participants self-reported reproductive and clinical history, including age at menarche, number of pregnancies, number of births, and menopausal status. Participants who self-reported a history of cancer and those missing this information were excluded from the present analysis, in addition to participants who were missing information on their menopausal status. Missing values for variables with <10% missing for both cases and controls were replaced with the median (for continuous variables) or modal (for categorical variables) value of their respective group. For variables with more than >10% missing, missing values were not imputed (age at menarche).

### Analytical approach

The sample was characterized via descriptive statistics, and results were reported as frequencies and proportions for categorical variables and medians (first quartile, third quartile) for continuous variables. Differences in associations by case/control status were tested using chi-square (*χ*^2^) tests or Fisher exact tests for categorical variables and Kruskal–Wallis nonparametric tests for continuous variables. We estimated the association between each lipid biomarker (total cholesterol, HDL, LDL, and triglycerides) and odds of BC using logistic regression models. Each measure was analyzed separately in the following three models: unadjusted, adjusted for age only, and adjusted for age, body mass index (BMI), age at menarche, number of pregnancies, number of births, hypertension at enrollment, and menopausal status. In a final model, we mutually adjusted for all lipid measures in addition to all previous covariates. Selection of covariates was based on a priori knowledge regarding the relationships between these factors and exposure and outcome. In each model, we specified each lipid biomarker as a categorical variable (high vs. low for total cholesterol, LDL, and triglycerides; and low vs. high for HDL), and also evaluated continuous measures of total cholesterol, HDL, LDL, and triglycerides based on one-unit SD increase (for total cholesterol, LDL, and triglycerides) or decrease (for HDL). We stratified our analysis of the continuous lipid profile measures by menopausal status. We further analyzed the subset of cases with cancer subtyping data available via multinomial logistic regression models. Control status was specified as the outcome reference group, and the fully adjusted model was repeated here to predict the odds of having Luminal A, Luminal B, TN, HER2 cancer subtypes. To address the issue of multiple comparisons, we applied the Bonferroni correction, and set significance at α = 0.0125 (0.05/4) for these associations. SAS v9.4 (SAS Institute, Cary, NC) was used for all analyses and significance was broadly set at α = 0.05.

### Ethical approval and consent to participate

All procedures performed in studies involving human participants were in accordance with the ethical standards of the institutional and/or national research committee and with the 1964 Helsinki declaration and its later amendments or comparable ethical standards. The study was approved by the Institutional Review Board of Duke University (Pro00102004). This article does not contain any studies with animals performed by any of the authors. Informed consent was obtained from all individual participants included in the study.

## Results

**Figure 1:**
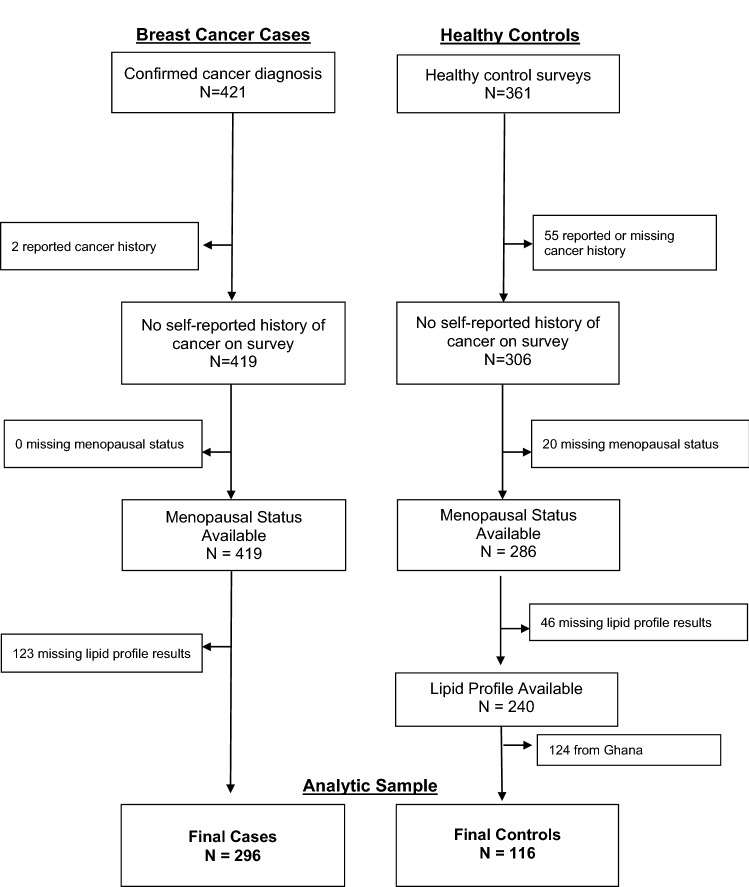
CONSORT diagram for MEND lipid profile analysis.

**Figure 2:**
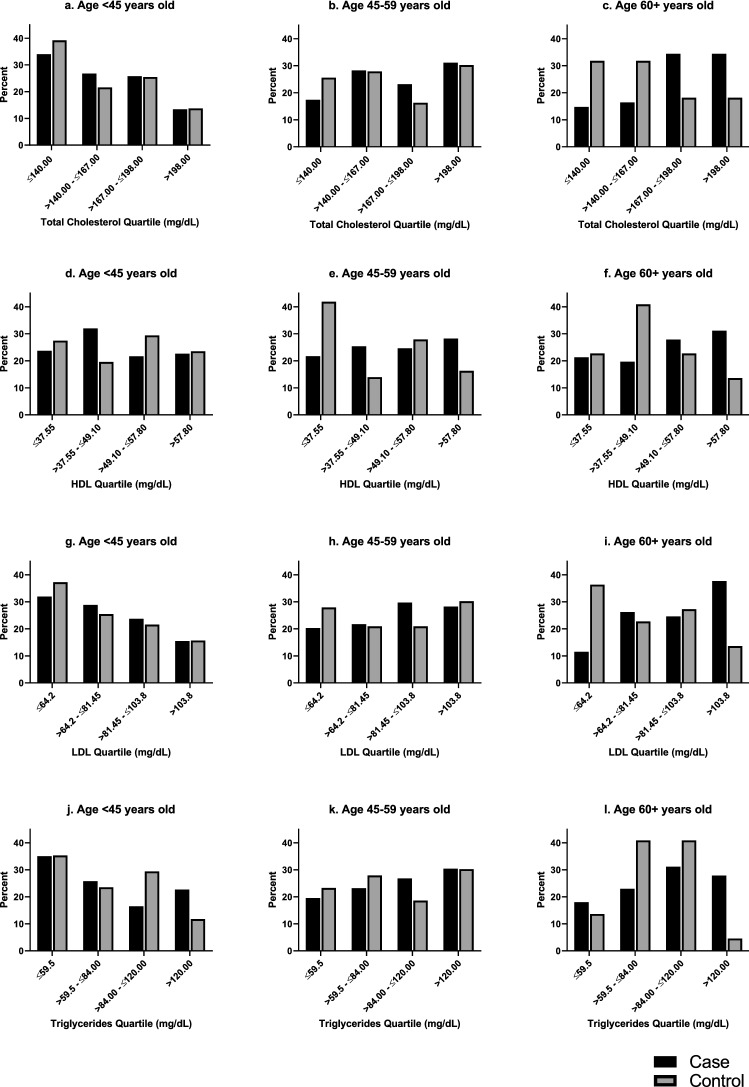
Lipid biomarker quartiles by case/control status and age.

The present analysis includes 296 BC cases and 116 healthy controls (Fig. 1). Cases were slightly older than controls—the median age at diagnosis for cases was 48.5 years, and the median age at enrollment for controls was 46 years (Table 1). Cases and controls were similar in terms of reproductive characteristics: number of pregnancies (5 vs. 5), number of births (4 vs. 4), and menopausal status (pre/peri-menopausal 48% vs. 49%). However, cases were more likely than controls to have high diastolic blood pressure (79.7 vs. 75.0), while controls were more likely to have higher BMI (25.4 vs. 26.5). Across total cholesterol quartiles (Table 2), those in the highest cholesterol group were older (*p* = 0.0003), and more likely to be a higher weight (*p* = 0.0593), have a higher blood pressure (systolic: *p* = 0.0067; diastolic: *p* = 0.0202) and be post-menopausal (*p* = 0.0003). A higher proportion of controls relative to cases were within the lowest total cholesterol quartiles among participants who were 60 years or older (Fig. 2).

**Table 1:** Clinical and reproductive characteristics of MEND breast cancer cases and controls.

Variable	Case N = 296	Controls N = 116
**Demographics**		
Age (years)^a^	48.5 (42.0, 57.0)	46.0 (40.0, 54.5)
**Clinical characteristics**		
Lipid Profile^a^		
Total cholesterol (mg/dL)	169.0 (142.5, 199.5)	162.0 (131.0, 190.0)
HDL-cholesterol (mg/dL)	49.6 (39.2, 59.2)	47.4 (35.7, 55.7)
LDL-cholesterol (mg/dL)	83.1 (66.6, 104.6)	76.2 (58.8, 97.5)
Triglycerides (mg/dL)	87.0 (60.0, 125.0)	74.0 (57.0, 104.0)
Total cholesterol (mg/dL)		
High (> 200)	73 (24.7)	24 (20.7)
Low (≤ 200)	223 (75.3)	92 (79.3)
HDL-cholesterol (mg/dL)		
Low (< 50)	151 (51.0)	65 (56.0)
High (≥ 50)	145 (49.0)	51 (44.0)
LDL-cholesterol (mg/dL)		
High (> 100)	89 (30.1)	27 (23.3)
Low (≤ 100)	207 (69.9)	89 (76.7)
Triglycerides (mg/dL)		
High (> 150)	47 (15.9)	12 (10.3)
Low (≤ 150)	249 (84.1)	104 (89.7)
Height (in)^a^	63.1 (61.4, 64.8)	63.0 (61.0, 65.4)
Weight (lb)^a^	143.0 (121.0, 165.2)	152.7 (127.9, 176.4)
Systolic BP^a^	125.0 (114.7, 140.5)	122.7 (109.2, 135.5)
Diastolic BP^a^	79.7 (70.7, 88.7)	75.0 (68.0, 82.8)
Body Mass Index (BMI)^a^	25.4 (22.2, 29.6)	26.5 (23.1, 31.4)
Hypertension at enrollment	87 (29.4)	25 (21.6)
**Reproductive history**		
Age at menarche		
≤ 13	60 (20.3)	19 (16.4)
> 13	230 (77.7)	76 (65.5)
Missing	6 (2.0)	21 (18.1)
Ever pregnant	282 (95.3)	110 (94.8)
Number of pregnancies^a,b^	5.0 (3.0, 6.0)	5.0 (3.0, 6.0)
Number of births^a,b^	4.0 (3.0, 5.0)	4.0 (2.0, 5.0)
Menopausal status		
Pre- or peri-menopause	143 (48.3)	57 (49.1)
Post-menopause	153 (51.7)	59 (50.9)
**Cancer Variables**		
Molecular Subtype	*N* = *124*	N/A^c^
Luminal A	33 (26.6)	
Luminal B	26 (21.0)	
Triple-negative	37 (29.8)	
HER2 +	28 (22.6)	
Grade		N/A^c^
1	5 (1.7)	
2	103 (34.8)	
3	58 (19.6)	
Unknown/Missing	130 (43.9)	

^a^Median (Q1, Q3).

^b^Among those who were ever pregnant.

^c^Cancer variables are not applicable to control participants.

**Table 2:** Clinical and reproductive characteristics of MEND cases and controls by quartile of total cholesterol.

Variable	Quartile of Total Cholesterol (mg/dL)				*P* value
	Q1 ≤140.00 mg/dL N = 104	Q2 >140.00–≤167.00 mg/dL N = 105	Q3 >167.00–≤198.00 mg/dL N = 102	Q4 >198.00 mg/dL N = 101	
**Case status**					0.1291
Case	66 (22.3)	75 (25.3)	78 (26.4)	77 (26.0)	
Control	38 (32.8)	30 (25.9)	24 (20.7)	24 (20.7)	
**Demographics**					
Age (years)^a^	44.0 (38.5, 52.0)	46.0 (41.0, 55.0)	49.0 (42.0, 59.0)	52.0 (47.0, 59.0)	**0.0003**
**Clinical characteristics**					
Height (in)^a^	63.0 (61.1, 64.9)	63.4 (62.2, 65.0)	63.0 (60.8, 64.6)	63.1 (61.6, 65.5)	0.3569
Weight (lb)^a^	137.7 (121.1, 160.7)	143.3 (120.8, 172.0)	143.3 (125.5, 174.4)	152.1 (130.1, 176.4)	0.0593
Systolic BP^a^	124.3 (111.0, 138.8)	119.7 (110.0, 134.0)	125.3 (115.7, 144.7)	130.3 (120.0, 145.0)	**0.0067**
Diastolic BP^a^	76.5 (69.7, 87.3)	75.0 (68.7, 82.3)	80.0 (70.7, 89.7)	80.0 (71.0, 90.0)	**0.0202**
Body Mass Index (BMI)^a^	24.6 (20.9, 28.7)	25.4 (21.8, 29.7)	25.9 (23.3, 30.3)	26.2 (23.1, 31.5)	**0.0396**
Hypertension at enrollment	26 (23.2)	20 (17.9)	29 (25.9)	37 (33.0)	**0.0385**
**Reproductive history**					
Age at menarche					**0.0222**
≤ 13	30 (38.0)	15 (19.0)	17 (21.5)	17 (21.5)	
> 13	65 (21.2)	81 (26.5)	79 (25.8)	81 (26.5)	
Missing	9 (33.3)	9 (33.3)	6 (22.2)	3 (11.1)	
Ever pregnant	97 (24.7)	101 (25.8)	96 (24.5)	98 (25.0)	0.5878
Number of pregnancies^a,b^	4.0 (4.0, 6.0)	5.0 (3.0, 7.0)	5.0 (3.0, 6.0)	5.0 (3.0, 6.0)	0.9480
Number of births^a,b^	4.0 (3.0, 5.0)	4.0 (3.0, 5.0)	4.0 (2.0, 5.0)	4.0 (2.0, 5.0)	0.8751
Menopausal status					**0.0003**
Pre- or peri-menopause	67 (33.5)	53 (26.5)	44 (22.0)	36 (18.0)	
Post-menopause	37 (17.5)	52 (24.5)	58 (27.4)	65 (30.7)	

^a^Median (Q1, Q3).

^b^Among those who were ever pregnant.

Where applicable, missing values were not used to compute p-value.

In fully adjusted multivariable logistic regression models (Table 3), one-unit SD increase in triglycerides was associated with 39% increased odds of BC overall (aOR: 1.39; 95% CI: 1.03, 1.86). Each SD increase in triglycerides remained significantly associated with odds of BC (aOR 1.47; 95% CI 1.06, 2.03) in the mutually adjusted model including all four lipid profile measures. Among post-menopausal women, one-unit SD increases in total cholesterol (aOR: 1.65; 95% CI: 1.06, 2.57), LDL cholesterol (aOR: 1.59; 95% CI: 1.04, 2.41), and triglycerides (aOR: 1.91; 95% CI: 1.21, 3.01) were associated with increased odds of BC in fully adjusted models. No significant associations were observed among pre/peri-menopausal women.

**Table 3:** Associations between lipid profile biomarkers and odds of cancer status.

	Model 1^a^ OR (95% CI)	Model 2^b^ aOR (95% CI)	Model 3^c^aOR (95% CI)	Model 4^d^aOR (95% CI)
**Total Cholesterol (mg/dL)**				
High vs. Low	1.26 (0.75, 2.11)	1.14 (0.67, 1.94)	1.13 (0.63, 2.02)	0.61 (0.28, 1.31)
Per one-unit SD increase	1.27 (1.00, 1.62)	1.23 (0.96, 1.57)	1.24 (0.94, 1.65)	0.65 (0.32, 1.33)
Pre/peri-menopausal	1.02 (0.78, 1.35)	0.99 (0.74, 1.32)	1.03 (0.73, 1.44)	0.63 (0.24, 1.66)
Post-menopausal	**1.77 (1.21, 2.61)**	**1.76 (1.19, 2.59)**	**1.65 (1.06, 2.57)**	0.67 (0.21, 2.12)
**HDL Cholesterol (mg/dL)**				
Low vs. High	0.82 (0.53, 1.26)	0.84 (0.54, 1.30)	0.94 (0.58, 1.52)	0.89 (0.54, 1.47)
Per one-unit SD decrease	0.81 (0.65, 1.01)	0.82 (0.66, 1.02)	0.89 (0.69, 1.14)	0.75 (0.52, 1.08)
Pre/peri-menopausal	0.91 (0.68, 1.21)	0.92 (0.69, 1.23)	0.88 (0.63, 1.23)	0.72 (0.43, 1.20)
Post-menopausal	**0.69 (0.49, 0.97)**	0.71 (0.50, 1.01)	0.81 (0.55, 1.18)	0.65 (0.37, 1.17)
**LDL Cholesterol (mg/dL)**				
High vs. Low	1.42 (0.86, 2.33)	1.30 (0.79, 2.16)	1.54 (0.87, 2.72)	2.32 (1.10, 4.89)
Per one-unit SD increase	1.23 (0.97, 1.56)	1.18 (0.93, 1.50)	1.29 (0.97, 1.72)	1.59 (0.88, 2.89)
Pre/peri-menopausal	1.02 (0.76, 1.37)	0.98 (0.73, 1.34)	1.03 (0.71, 1.49)	1.36 (0.57, 3.23)
Post-menopausal	**1.57 (1.08, 2.28)**	**1.55 (1.06, 2.25)**	**1.59 (1.04, 2.41)**	1.76 (0.73, 4.27)
**Triglycerides (mg/dL)**				
High vs. Low	1.64 (0.83, 3.21)	1.54 (0.78, 3.04)	1.61 (0.76, 3.39)	1.70 (0.79, 3.66)
Per one-unit SD increase	**1.32 (1.03, 1.70)**	1.28 (0.99, 1.64)	**1.39 (1.03, 1.86)**	**1.47 (1.06, 2.03)**
Pre/peri-menopausal	1.19 (0.82, 1.72)	1.15 (0.79, 1.67)	1.09 (0.73, 1.64)	1.24 (0.77, 2.00)
Post-menopausal	**1.45 (1.02, 2.06)**	**1.47 (1.03, 2.10)**	**1.91 (1.21, 3.01)**	**2.07 (1.25, 3.43)**

Logistic regression models  for the odds of having cancer by lipid profile biomarkers. ORs per one-unit SD were modeled as a one-unit increase/decrease in SD of the lipid profile variable from its mean-centered value. Bolded values indicate significance at p<.05.

High total cholesterol defined as >200 mg/dL; low HDL defined as <50 mg/dL; high LDL defined as >100 mg/dL; high triglycerides defined as >150 mg/dL.

^a^Model 1, unadjusted.

^b^Model 2, adjusted for age.

^c^Model 3, additionally adjusted for clinical characteristics: BMI, age at menarche, number of pregnancies, number of births, hypertension at enrollment, and menopausal status.

^d^Model 4, additionally adjusted for all lipid profile biomarkers: total cholesterol, LDL, HDL, and triglycerides.

*OR* odds ratio, *CI* confidence interval, *aOR* adjusted odds ratio, *SD* standard deviation.

In multinomial logistic regression models predicting the odds of each molecular subtype relative to controls (Table 4), clinically low HDL was associated with 2.7 times the odds of TNBC (aOR: 2.67; 95% CI: 1.10, 6.49). Additionally, each unit SD increase in LDL was associated with 64% increased odds of Luminal B BC (aOR: 1.64; 95% CI: 1.06, 2.55). These associations were both significant at α = 0.05 without accounting for multiple comparisons; however, they were not significant following the Bonferroni correction (α = 0.0125). Among post-menopausal women, one-unit SD increases in LDL cholesterol and triglycerides were significantly associated with increased odds of Luminal B BC (aOR: 3.52; 95% CI: 1.48, 8.35) and HER2 BC (aOR: 4.15; 95% CI: 1.71, 10.05), after accounting for multiple comparisons. No significant associations were observed among pre/peri-menopausal women in the subtype analysis.

**Table 4:** Associations between lipid biomarkers and breast cancer subtype.

	Luminal A	Luminal B	Triple Negative	HER2
	aOR (95% CI)	aOR (95% CI)	aOR (95% CI)	aOR (95% CI)
**Total Cholesterol (mg/dL)**				
High vs. Low	0.63 (0.20, 1.98)	1.38 (0.49, 3.92)	0.92 (0.35, 2.41)	1.17 (0.41, 3.31)
Per one-unit SD increase	0.99 (0.64, 1.55)	1.34 (0.92, 1.96)	0.98 (0.63, 1.51)	1.01 (0.65, 1.57)
Pre/peri-menopausal	0.91 (0.54, 1.54)	1.12 (0.75, 1.66)	0.77 (0.42, 1.42)	0.80 (0.45, 1.44)
Post-menopausal	1.25 (0.50, 3.14)	2.31 (0.87, 6.11)	1.32 (0.63, 2.77)	1.82 (0.64, 5.24)
**HDL Cholesterol (mg/dL)**				
Low vs. High	0.92 (0.38, 2.22)	1.11 (0.44, 2.78)	**2.67 (1.10, 6.49)**	0.91 (0.37, 2.26)
Per one-unit SD decrease	0.96 (0.64, 1.44)	0.84 (0.53, 1.32)	1.49 (0.94, 2.34)	0.93 (0.60, 1.45)
Pre/peri-menopausal	0.86 (0.51, 1.44)	0.89 (0.50, 1.57)	1.23 (0.66, 1.95)	0.93 (0.54, 1.61)
Post-menopausal	0.80 (0.34, 1.87)	0.65 (0.27, 1.57)	1.81 (0.86, 3.77)	0.69 (0.25, 1.88)
**LDL Cholesterol (mg/dL)**				
High vs. Low	1.96 (0.71, 5.40)	2.56 (0.92, 7.11)	2.10 (0.87, 5.11)	1.72 (0.61, 4.80)
Per one-unit SD increase	1.21 (0.75, 1.98)	**1.64 (1.06, 2.55)**	1.34 (0.88, 2.06)	1.02 (0.60, 1.72)
Pre/peri-menopausal	0.88 (0.47, 1.64)	1.10 (0.68, 1.76)	1.00 (0.61, 1.64)	0.72 (0.35, 1.46)
Post-menopausal	1.59 (0.63, 4.00)	3.52 (1.48, 8.35)*	1.85 (0.87, 3.91)	1.37 (0.54, 3.46)
**Triglycerides (mg/dL)**				
High vs. Low	1.03 (0.25, 4.34)	1.12 (0.28, 4.58)	1.57 (0.51, 4.85)	2.64 (0.81, 8.59)
Per one-unit SD increase	1.32 (0.81, 2.15)	1.53 (0.97, 2.42)	1.38 (0.90, 2.11)	1.36 (0.85, 2.18)
Pre/peri-menopausal	1.20 (0.65, 2.21)	1.26 (0.69, 2.30)	0.84 (0.41, 1.74)	0.67 (0.28, 1.61)
Post-menopausal	1.49 (0.60, 3.73)	1.84 (0.87, 3.88)	**2.27 (1.16, 4.43)**	4.15 (1.71, 10.05)*

Multinomial logistic regression models for the odds of having each cancer subtype, compared to no cancer, by lipid profile biomarkers. ORs per one-unit SD were modeled as a one-unit increase/decrease in SD of the lipid profile variable from its mean-centered value.

Bolded values indicate significance at *p*<.05.

* indicates significance at *p*<.0125.

High total cholesterol defined as >200 mg/dL; low HDL defined as <50 mg/dL; high LDL defined as >100 mg/dL; high triglycerides defined as >150 mg/dL.

Models were adjusted for age and clinical characteristics: BMI, age at menarche, number of pregnancies, number of births, hypertension at enrollment, and menopausal status.

*aOR* adjusted odds ratio, *CI* confidence interval, *SD* standard deviation.

## Discussion

For the first time, we describe the results of a case-control analysis of lipid biomarkers (total cholesterol, HDL cholesterol, LDL cholesterol, and triglycerides) and odds of BC and molecular subtypes among African women. Among cases and controls, those who were older, had high BMI and high blood pressure at enrollment were more likely to have high cholesterol. Higher triglycerides were associated with increased odds of BC in fully adjusted models. Among post-menopausal women, higher total cholesterol, LDL cholesterol, and triglycerides were all associated with increased odds of BC. In the analysis of molecular subtypes, low HDL and high LDL were associated with increased odds of the TNBC and Luminal B subtypes, respectively. Among post-menopausal women, higher LDL and triglycerides were significantly associated with increased odds of the Luminal B and HER2 subtypes, respectively.

Several past studies among populations from the US, Europe, and Asia have evaluated the association between lipid biomarkers and BC risk, however results have been inconsistent. For total cholesterol, one study in Korea noted a positive association with BC risk^26^, but others in the US and Europe, like ours, have found no association^16,27^, and one study additionally observed an inverse association^28^. In the context of LDL, a case-control study among African American women in the US found a 59% reduction in risk among those who had clinically high levels of LDL cholesterol^15^. Other studies in the US, Asia, and Europe, like ours, have also found no association^14,29^, although one Mendelian randomization study among those of European descent documented a positive association^30^. We did not observe a significant association between HDL cholesterol and odds of BC. One study in Europe found an inverse association between HDL cholesterol and BC risk^28^, while a Mendelian randomization analysis in Europe found that an increase in genetically-predicted HDL was associated with increased BC risk^31^. However, others in the US and Europe have failed to find an association with HDL^29,32^. Regarding triglycerides, one study using the Swedish AMORIS database noted a weak protective association with risk of BC^33^, while others still have reported no association^28,34^. On the contrary, two small case–control studies in India and the US, like ours, found a positive association between triglycerides and BC^35,36^. Ultimately, there is inconclusive evidence regarding the role of lipid biomarkers in BC risk, suggesting that additional studies on this topic are still warranted, and importantly, studies from diverse populations will be needed to determine if region-specific associations may explain the disparate findings.

Our analysis of the association between lipid measures and BC subtypes revealed that low HDL cholesterol level is associated with increased odds of TNBC, and that higher LDL is associated with increased odds of Luminal B BC. We also found that higher LDL is associated with increased odds of Luminal B BC and that higher triglycerides is associated with increased odds of HER2 BC among post-menopausal women, but not among pre/peri-menopausal women. Our results are inconsistent with findings from a study from Korea reporting that low HDL cholesterol and high triglycerides were associated with an increased risk of developing hormone receptor negative tumors among premenopausal women^17^. Consistent with our results, a study among patients from the US found that dyslipidemia, investigated as part of metabolic syndrome, was associated with TNBC, and specifically, low HDL was associated with TNBC^37^. Given that epidemiologic studies evaluating the association of lipid biomarkers and BC subtypes are very limited, our findings provide important initial evidence upon which future studies can expand.

The biological mechanisms underlying the association between lipids and BC remain unclear and is an active area of research. Studies have suggested that elevated serum cholesterol levels may advance tumor progression^38^, and a recent review of laboratory studies suggests that cholesterol is capable of regulating proliferation, migration, and signaling pathways in BC^39^. Research on mechanisms underlying risk by molecular BC subtype is limited, however, as suggested by Llanos et al.^15^, it is possible that HDL influences overall BC risk by moderating biologically active estradiol^40^, a risk factor for BC among postmenopausal women^41^. Low HDL cholesterol may reflect an unfavorable hormonal profile, and the conversion of androgens to estrogens within adipose tissues may represent a causal mechanism for the inverse association between HDL and BC risk^40^. Fernandez and Murillo demonstrated that HDL is inversely correlated with waist circumference and higher BMI^42^, providing support for the mediating role of adiposity. In a previous study of the same population, we found that higher BMI was associated with reduced odds of breast cancer^43^. It is also possible that HDL plays a key role in reverse cholesterol transport that may contribute to the blocking of tumor progression and ultimately BC incidence. Although reverse cholesterol transport may be its primary role, HDL has also been shown to possess antimicrobial, antioxidant, antiglycation, anti-inflammatory, antiatherogenic, and immunosuppressive properties^44–46^. The numerous functions of HDL provide a plethora of opportunities for novel research, but also make pinpointing the exact mechanism by which it may confer protection against BC difficult. Some of the conflicting results in the epidemiology of HDL and BC risk may be explained, in part, by the observation that the environment in which HDL exists in the body may influence its effect on BC cells. Pan and colleagues used in vivo and in vitro models of BC and observed that oxidized HDL and HDL derived from diabetic patients were associated with the promotion of metastasis and invasion to surrounding tissues^47–49^. Future studies on the role of cholesterol oxidation products and signaling pathways may shed additional insights into these mechanisms^50,51^. Still, these explanations are not specific to TNBC, and further studies are needed to fully characterize these mechanisms by BC subtype.

Understanding the mechanism by which HDL has shown an inverse association with TNBC is further complicated by challenges related to sample size as TNBC typically accounts for an estimated 15–20% of all BCs. Further, although an estimated 80% of TNBC are classified as the basal BC intrinsic subtype^52^, new research suggests that TNBC may actually be quite heterogenous with respect to cellular and molecular features^53^. African American women tend to demonstrate patterns of TNBC occurrence that map more closely with women from western and sub-Saharan Africa than they do with women from east Africa, implicating a role of genetic factors^54,55^. That clinically low HDL was associated with TNBC provides a possibility of a therapeutic target for the BC subtype that is the most aggressive, has a poor prognosis, and by definition, cannot be targeted with pharmaceutical therapy designed for ER + cancers. Still as we point out, the mechanisms underlying the inverse association between HDL and TNBC risk require vigorous investigations, perhaps pooled analyses across existing studies may provide additional insight.

There are several strengths and limitations of this study that may impact the interpretation of these results. Many covariates were self-reported by participants, potentially introducing recall bias into our analysis. However, our main exposures of interest, namely total cholesterol, HDL, LDL, and triglycerides were assayed for cases and controls at the same time following the same standard assay protocol, thus minimizing batch effects. Moreover, due to the case-control study design, we are unable to rule out the possibility of reverse causality. It is possible that lipid levels may be influenced by the presence of BC, producing the observed association. Furthermore, due to lack of available data, we were unable to incorporate the use of drug treatment for dyslipidemia and hypertension in our analysis; it is possible that treatment for these conditions may influence results. We were also unable to adjust our models for waist circumference, as this variable was not available for controls. Although we adjusted for BMI, we acknowledge that there may be residual confounding by adiposity. In future studies, we additionally hope to evaluate potential confounding by socioeconomic status. Strengths of our study include the use of histologically confirmed cancer cases and pathologically verified molecular subtypes assessed by a single pathologist, the availability of data on critical reproductive history and clinical characteristics for covariate adjustment, and the unique study population of Nigerian women—adding to the diversity of study populations for this topic. Although our sample size is modest compared with other large cohorts, we emphasize that our study is the first to characterize the association between lipid profile measures and BC risk in Nigeria, and one of very few studies worldwide to evaluate the association between lipid profile measures and BC risk by subtype. We lay important groundwork for future large prospective studies among African women.

## Conclusions

We evaluated the association between lipid profile biomarkers and odds of BC for the first time among Nigerian women, a population that is disproportionately affected by aggressive TNBC. Past research on this topic is highly conflicting, and few studies worldwide have evaluated associations by specific BC molecular subtypes. We report a positive association between triglycerides and odds of BC, and between low HDL with TNBC, and high LDL with Luminal B BC. Lipids are easily measured in clinical settings, making this an attractive target for cancer prevention strategies that may reduce the risk of BC among Nigerian women.

## Data Availability

The datasets used and/or analyzed during the current study are available from the corresponding author on reasonable request.

## Abbreviations

95% CI: 95% Confidence interval
aOR: Adjusted odds ratio
BMI: Body mass index
BC: Breast cancer
CKD: Chronic kidney disease
ER: Estrogen receptor
H3A: Human Heredity and Health Africa
HDL: High-density lipoprotein cholesterol
HER2: Human epidermal growth factor-2
IHC: Immunohistochemistry
LDL: Low-density lipoprotein cholesterol
MEND: Mechanisms for Established and Novel Risk Factors for Breast Cancer in Women of Nigerian Descent
OR: Odds ratio
PR: Progesterone receptor (PR)
SD: Standard deviation
TME: Tumor microenvironment
TN: Triple-negative
US: United States
